# Monitoring of DDT in Agricultural Soils under Organic Farming in Poland and the Risk of Crop Contamination

**DOI:** 10.1007/s00267-020-01347-9

**Published:** 2020-08-19

**Authors:** Eligio Malusá, Małgorzata Tartanus, Witold Danelski, Artur Miszczak, Ewelina Szustakowska, Joanna Kicińska, Ewa M. Furmanczyk

**Affiliations:** Research Institute of Horticulture, Skierniewice, Poland

**Keywords:** Contamination source, DDT metabolites ratio, DDT monitoring, Plant residues uptake, Soil pollution

## Abstract

The analysis of 142 agricultural soil samples collected in organic farms across Poland with the intent to evaluate the level of DDT contamination resulted in more than 80% of the soils containing DDT. The ΣDDT (sum of all metabolites and isomers) concentration ranged between 0.005 and 0.383 mg/kg ΣDDT, with an average value of 0.064 mg/kg ΣDDT. However, the majority of plant samples collected from the crops growing on the sampled soils did not contain detectable DDT residues. The high DDT pollution levels detected in samples from four voivodeships (regions) among those monitored have been hypothesised to be linked to horticultural productions occurring to the sampled fields and typical of those regions, particularly in big-sized farms, during the period of DDT application, as well as the number of pesticides landfills present in these voivodeships. The elaboration of the o,p′-DDT/p,p′-DDT and DDT/(DDE + DDD) ratios to appraise the source or the period of contamination suggested that the contamination originated from past use of DDT rather than from impurities of more recent applications of other formulated substances. Such outcome thus suggests that the risk of contamination of organic products is likely derived from general environmental pollution levels rather than from the use of unauthorised substances in organic farming productions. Data from a trial with artificial contamination of soils indicated that using the DDT/(DDE + DDD) ratio in the presence of a low level of contamination could be less reliable than in highly contaminated soils.

## Introduction

The extensive and widespread use of plant protection products (PPPs) in the management of agricultural crops has resulted in their increasing presence in agroecosystems, thereby raising environmental, health and food safety concerns. To reduce the negative impact of PPPs, the implementation of integrated pest management practices has been promoted since the 1950s (Boller et al. 2006) and is now compulsory in the entire European Union (EU). In parallel, the conversion to organic farming pursues a limited use of PPPs, forbidding the application of all the synthetic ones (Matyjaszczyk 2018). However, the presence of residues in the soil deriving from previous, long-term applications of PPPs, particularly those having a long persistence, could pose risks of contamination of products certified as organic (EFSA European Food Safety Authority 2018a).

1,1,1-Trichloro-2,2-bis(4-chlorophenyl) ethane (DDT) was among the organochlorine pesticides mainly used in agriculture since the 1940s. It was also applied to reduce the occurrence of mosquito-transferred malaria, and has been classified as a persistent organic pollutant (Hung et al. 2016). Depending on soil microbial activity (Pan et al. 2016), DDT could undergo slow metabolisation into DDE or DDD, which are also very persistent in soil and harmful to human health. DDT usage has been banned in Europe since the 70s, in Poland in 1976, but in the 1990s another compound, Dicofol (acaricide), synthesised from technical DDT and containing this compound as an impurity, was applied on crops. Its use could thus have also contributed as a possible new source of DDT contamination in the environment (Qiu and Zhu 2010). Studies on the presence of different DDT isomers and metabolites pointed out that some ratios between them could be useful to determine the likely period of soil contamination, thus allowing to discriminate between ‘old’ and ‘more recent’ paths of soil pollution (Iwata et al. 1993; Qiu and Zhu 2010).

In recent years, monitoring data from different countries all over the world has been reported about the distribution and level of contamination with different POPs, including DDT (Tieyu et al. 2005; Škrbić and Durišić-Mladenović 2007; Arias et al. 2011). In the EU, several studies have reported data from specific regions, for example: Northern France (Villanneau et al. 2011), the Almeria province in South-Eastern Spain (Plaza-Bolaños et al. 2012), different regions of Italy (Qu et al. 2016; Thiombane et al. 2018), North-Eastern Romania (Tarcau et al. 2013), and central Germany (Manz et al. 2001). All these studies clearly pointed out the quite high frequency of DDT contamination in agricultural soils. Even though DDT content varied significantly depending on the history of land use, source of contamination or other environmental factors, most of the available data are strongly suggesting that the contamination in several countries is a result of historical application rather than recent use (Tieyu et al. 2005; Škrbić and Durišić-Mladenović 2007; Plaza-Bolaños et al. 2012; Thiombane et al. 2018; Silva et al. 2019).

DDT soil contamination in Poland was pointed out in the results of a general monitoring programme (GIOŚ Main Inspectorate of Environmental Protection 2015) and confirmed by a previous study (Tartanus et al. 2017) as well as by a recent survey on arable soils (Ukalska-Jaruga et al. 2020).

Despite the availability of data about the occurrence of DDT residues in European arable soils, information for soils managed according to organic farming rules is scant. Nevertheless, the analysis of EU pesticide residues monitoring programme data pointed out that organic produce can be contaminated with pesticides, including DDT (EFSA European Food Safety Authority 2018a). Therefore, in an effort to verify the level and sources of contamination of organic certified products and to assess the risk of not only unauthorised uses—also in compliance with the recommendations of the European Food Safety Agency (EFSA European Food Safety Authority 2018b)—we have conducted a targeted assessment of DDT contamination in Polish soils from fields managed according to organic farming methods (i.e., certified according to EU Commission Regulation EC 2008). A second objective was to assess the likelihood of finding DDT residues in the crops grown on the sampled soils, trying also to verify the possible origin of the contamination. Here we present the findings of this monitoring programme together with the results of trials performed to evaluate the kind of contamination.

## Materials and Methods

### Soil and Plant Sampling

Soil samples were collected from 142 fields (one sample per field, according to the method described below), managed and certified according to EU organic farming rules, located in 15 Polish voivodeships (regions), in 2016 and 2017, always in the period from mid-September to mid-October (end of summer season), to account for and avoid seasonality variation as well as to assess the possibility of unauthorised uses. Each field was identified by a code with the name of the location (town/village), which it was administratively belonging from. Sometimes, in the same location several different fields were sampled, belonging to the same or different farms: in this case the same name is followed by a different number, to allow sample identification. The selection of the fields for sampling was based on the following criteria: farms growing horticultural crops (fruits and vegetables), frequency of organic farms in a given region, farm location in rural area (i.e., not close to possible industrial sites of pollution). Soil sampling methodology followed the method from the Polish Regulation ‘on the sampling of plants, plant products or objects for testing for residues of PPPs’ (Polish Official Gazette 2013) in order to assure the results complying with the national legislation due to their relevance to the field certification. For the same reason, the classification of the DDT content followed the Polish Regulation ‘on the standards of evaluation of surface soil contamination’ (Polish Official Gazette 2016). Soil subsamples were collected with an Egner’s sampler from a depth of 0–25 cm from about 20–25 randomly distributed points within each selected field (up to 1 ha). These subsamples were pooled and mixed to form the composite sample of about 1 kg of soil delivered to the laboratory. The samples were frozen and stored until analysis.

To evaluate the bioaccumulation capacity of the plants growing on the sampled soils, samples of about 300 g of different plant organs (aboveground and roots) were randomly collected from each crop present on the sampled field according to the official Polish method (Polish Official Gazette 2013). The plant tissues were cleaned from soil residues, washed with water, pre-cooled, comminuted, homogenised in the presence of dry ice and stored frozen until analysis.

The pH of the soil samples was determined on KCl extract: 10 g of homogenised and air-dried soil was mixed with 25 ml of 1 M KCl, and the measure was done on the solution after 24 h with a pH metre (Fisher Scientific Poland) (Gorlach and Mazur 2002).

### Artificial Contamination of Soil

To verify the validity in classifying the contamination period of the soil according to the DDT/(DDE + DDD) ratio, an experiment with artificial contamination of soils was performed. A soil originating from a field that, in several preliminary analyses, did not show any contamination with DDT, and a soil with detectable DDTs concentration (see ‘Results’) were spiked in with analytical grade p,p′-DDT (Sigma Aldrich). A stock solution of DDT in acetone was mixed with water (3:97 v/v) to prepare the working solution applied to the soils. The DDT solution was uniformly sprayed using a hand sprayer onto 10 l of homogenised soil arranged as a 2-cm-thick layer over a plastic film, in a quantity necessary to deliver 0.125 or 0.200 mg of analytical grade p,p′-DDT per liter of soil. The soil was then placed into pots keeping it in the plastic film to avoid the possibility of contact of the soil with the pot, and kept in a cold greenhouse for about 5 months. The procedure was performed individually for the soil of each pot (40 pots in total) that was subsequently used for analysis. Sampling of this soil was performed collecting about 10–12 soil subsamples at random and pooled to form the composite sample of about 200 g of soil delivered to the laboratory. The samples were frozen and stored until analysis.

### Analytical Determination of DDT and of its Metabolites

Residues determination of DDT isomers and metabolites (p,p′-DDT, o,p′-DDT, p,p′-DDD, o,p′-DDD, o-p′-DDE, p-p′-DDE) in soil and plant material was made by gas chromatography (Agilent Technologies 6890N), using a Zebron™ ZB-MultiResidue™-1 chromatographic column, with mass detector (5975B Inert XL MSD), following the manufacturer’s working conditions, according to ISO 17025 and GLP standards.

Extraction of the compounds was carried out according to the QuEChERS method (EN 15662:2008). About 10 g (soil, fruits) or 5 g (roots, stems, leaves) aliquots of the stored samples were utilised for the extraction procedure. The sample was extracted by shaking the materials for 3 min using the QuEChERS Hand Motion Shaker (Eberbach model EL680.Q.25 QuEChERS) with a solution containing 10 ml water, 10 ml acetonitrile (LC/MS grade, Merck), 4 g magnesium sulfate, 1 g sodium chloride, 1 g trisodium citrate dihydrate and 0.5 g disodium hydrogen citrate sesquihydrate (all reagents included in the QuEChERS Extraction Kit, Agilent Technologies). The suspension was centrifuged (MPW Med. Instruments) for phase separation at 8000 rpm for 5 min at room temperature. An aliquot of the organic phase (~2 ml) was transferred to Eppendorf safe lock tubes, cleaned-up by dispersive solid-phase extraction with 25 mg of amino sorbent (PSA) and 150 mg of magnesium sulphate (both included in Dispersive SPE 2 ml, Fruits & Veg EN, Agilent Technologies) for removal of residual water and centrifuged at 8500 rpm for 1 min at room temperature. An aliquot of 1.0 ml was transferred to a chromatographic vial and 0.1 ml of Triphenyl phosphate solution (Dr Ehrenstorfer) was added as an internal standard. Isomers and metabolites content was evaluated by comparing the retention time of certified analytical standards (p,p′-DDT purchased from Sigma Aldrich, the other compounds from Dr Ehrenstorfer), taking into account specific matrix effects, corrected by the internal standard. Data obtained from plant samples were adjusted to fresh mass, while those for soil samples were adjusted to dry mass (soil drying was obtained by heating at 80 °C till constant weight was recorded).

The control of the method was performed carrying out ten analyses (five for each fortification level) of soil samples fortified with 0.010 mg/kg and 0.100 mg/kg of DDT and its metabolites for each isomer separately. Average recovery was 93–110% (SD 7–17%), and 85–102% (SD 10–17%) for concentrations of 0.010 mg/kg and 0.100 mg/kg, respectively. The limit of determination was 0.003 mg/kg.

### Statistical Analysis

Statistical analysis of data was performed using the R software version 3.5.0 (R Core Team 2019). The Shapiro–Wilk test was used to verify if the data followed a normal distribution and the Levene’s test was used to verify the homogeneity of variances. In case of not normal distribution, the non-parametric Kruskal–Wallis analysis with Dunn’s post hoc test with Benjamini–Hochberg correction was used with significance set at *p* ≤ 0.05. The Pearson correlation coefficient was calculated using rcorr function from the ‘Hmisc’ package of the R software.

## Results and Discussion

### DDT Occurrence in Polish Soils

**Fig. 1:**
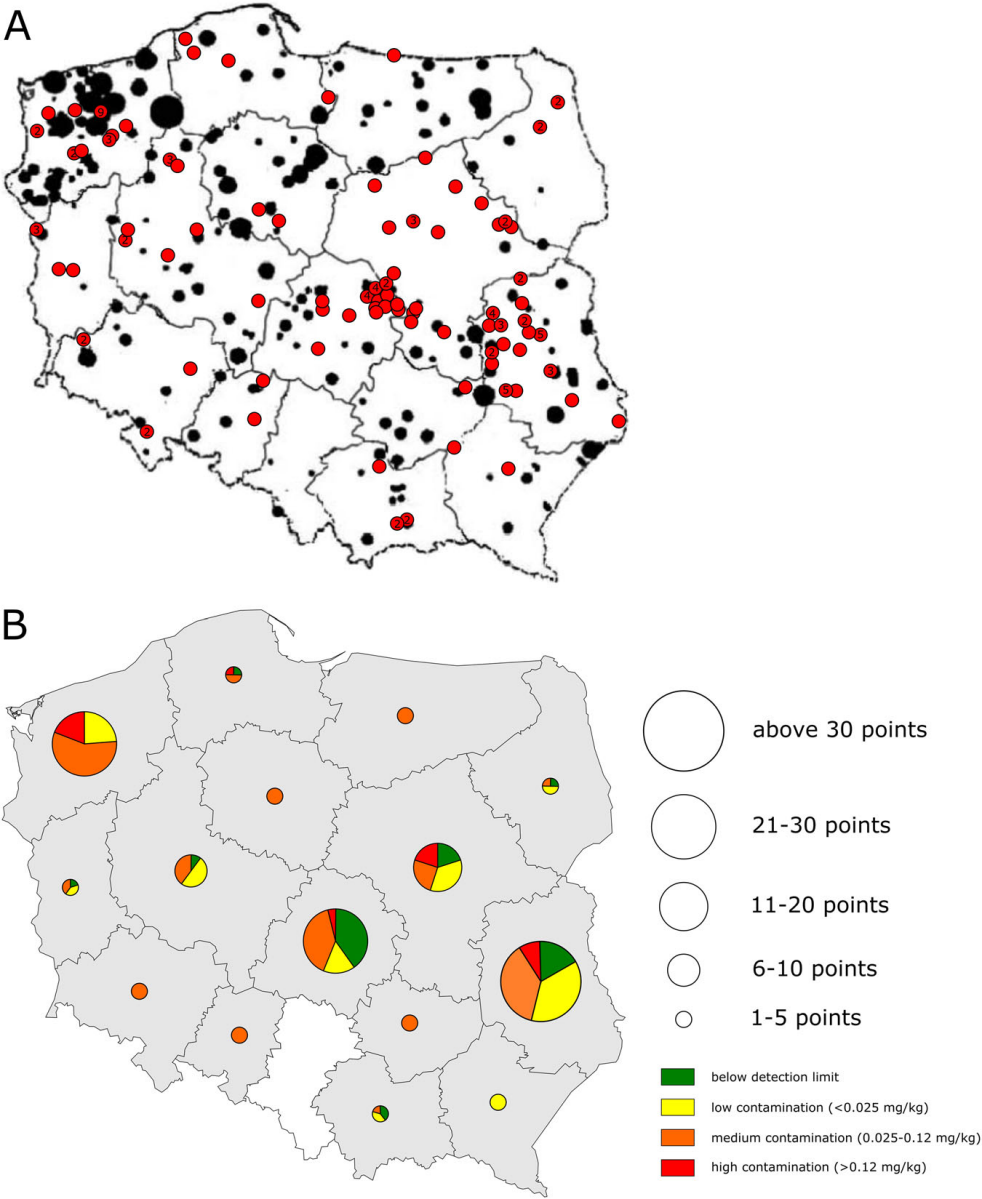
The distribution of DDT monitoring sites and level of soil contamination in the different voivodeships of Poland. **a** The location of the sampled sites is overlapped to the distribution of pesticide dumps (adapted from Witczak et al. 2013). Red circles represent the sampling locations (the numbers in some of them refer to the number of sampling sites), black circles represent the dump locations. **b** The level of DDT contamination is shown by pie charts where size corresponds to the number of sampled localisations in the corresponding voivodeship. The pie charts represent the proportions of the soil samples with different level of contamination, as specified in the legend

The results of the analysed soil samples collected from fields managed according to organic farming practices are summarised on Fig. 1 and fully reported in Table EMS1 (Online Resource). Only 26 soils out of the 142 sampled (comprising 18.31% of all samples) were free from detectable DDT residues. They were located in eight voivodeships. The rest of the investigated fields (81.69%) contained DDT or its metabolites at levels ranging from 0.005 to 0.383 mg/kg. The highest concentration was found in a sample from a soil in Krukowo (Masovian Voivodeship—Central Poland).

Considering that according to Polish law (Polish Official Gazette 2016) the level of DDT and its metabolites for agricultural soils should not exceed 0.120 mg/kg and that four contamination categories are foreseen, we have classified the analysed samples into the same categories, as follows: contamination below the detection limit (DDT was not detected), low contamination (detected concentration up to 0.025 mg/kg), medium contamination (0.025–0.120 mg/kg) and high contamination (>0.120 mg/kg). Most of the samples were contaminated at medium level (42.96%), with an average (arithmetic mean) content of 0.064 mg/kg of ΣDDT. However, thirteen locations (representing 11% of the contaminated fields) showed a high contamination. These fields were from five voivodeships: Masovian (four fields), West Pomeranian (four fields), Lublin (three fields), Łódź and Pomeranian (each with one field).

These results are partially in line with the outcomes of a previous environmental monitoring programme carried out on arable fields in Poland (Ukalska-Jaruga et al. 2020). Indeed, 100% of the 216 sampled locations resulted contaminated with DDT, 55% of them contaminated at medium level (according to our classification), and with a wider range (from 0.006 to 0.485 mg/kg), but showing similar average amounts of ΣDDT (0.045 mg/kg) in comparison to the results of the present work (Ukalska-Jaruga et al. 2020). The comparison is thus suggesting that fields managed according to organic farming practices are contaminated by DDT residues to a lesser extent than other arable areas. A previous initial assessment of soil DDT contamination in organic farms carried out in a limited number of fields also indicated a similar result (Tartanus et al. 2017). Such outcome could be explained considering the biological fertility of the soils under conventional or organic management. Soil contamination with pesticides has been proved to decrease both microbial biodiversity and functionality (Jiao et al. 2016; Borowik et al. 2017), which could in turn influence the decomposition of the residues. On the other hand, organic farming is based on practices of soil management that are meant to improve its fertility and they have been found to increase microbial biodiversity and activity (Maeder et al. 2002). Therefore, such contrasting trends in conventional and organically managed soils could in the long term affect also the persistence of pesticide residues, particularly of POPs such as DDT, supporting a steady reduction in case of organically managed fields.

The amount of the different metabolites and isomers of DDT differed widely between the sampled fields. Among the analysed compounds o,p′-DDE was always below the detection limit, and o,p′-DDT was identified only in three samples of the high contamination group. Both DDD isomers (o,p′-DDD and p,p′-DDD) were determined mainly in medium and highly contaminated localisations (47 and 110 sites, respectively; Table EMS1, supplemented as an Online Resource).

The detection of DDT in the majority of the monitored soil samples strongly confirms the high persistence of this compound in soil (Lewis et al. 2016) and a relatively diffused contamination of Polish agricultural soils (Ukalska-Jaruga et al. 2020), similar to several agricultural areas across many European countries (Silva et al. 2019). Besides contamination due to the use of DDT as an insecticide, another possible source of this diffused contamination could derive from underground landfills of pesticides (widely known as ‘tombs’) that were created in Poland from 1965 up to the early 1990s where about 200,000 kg of pesticides were buried (Gałuszka et al. 2011). Many of these landfill sites were simple excavations without any isolation from the surrounding ground, located in highly permeable sandy soils, frequently built close to water reservoirs and agricultural areas, thus likely to pollute the environment via groundwater (Siłowiecki 2002; Ignatowicz 2009), which effect remained observable also in derived food products like milk (Witczak et al. 2013). The content of a pesticide tomb was dominated by organochlorine pesticides, with DDT as the prevailing compound (Gałuszka et al. 2011). However, we could not verify such hypothesis not knowing their precise location.

The high incidence of DDT residues in Polish agricultural soils can also be linked to the establishment of huge state-managed farms after World War II. These farms were characterised by high use of pesticides and at the end of 1980s they still covered about 24% of Polish agricultural land (Bański 2011), when DDT use was already banned (gradually withdrawn since 1972 and fully terminated in 1976). Moreover, these farms favoured monoculture, which has been proved to negatively influence soil biodiversity (Li et al. 2019), thereby limiting the natural biodegradation potential of soil environment. However, potential air-borne soil contamination due to the past production of DDT in Poland (Niewiadomska and Zmudzki 2011) and nearby countries (e.g., Czech Republic) could also be accounted for the widespread presence of DDT in Polish soils as found in other Eastern Europe and Balkans countries (Růžičková et al. 2008).

**Fig. 2:**
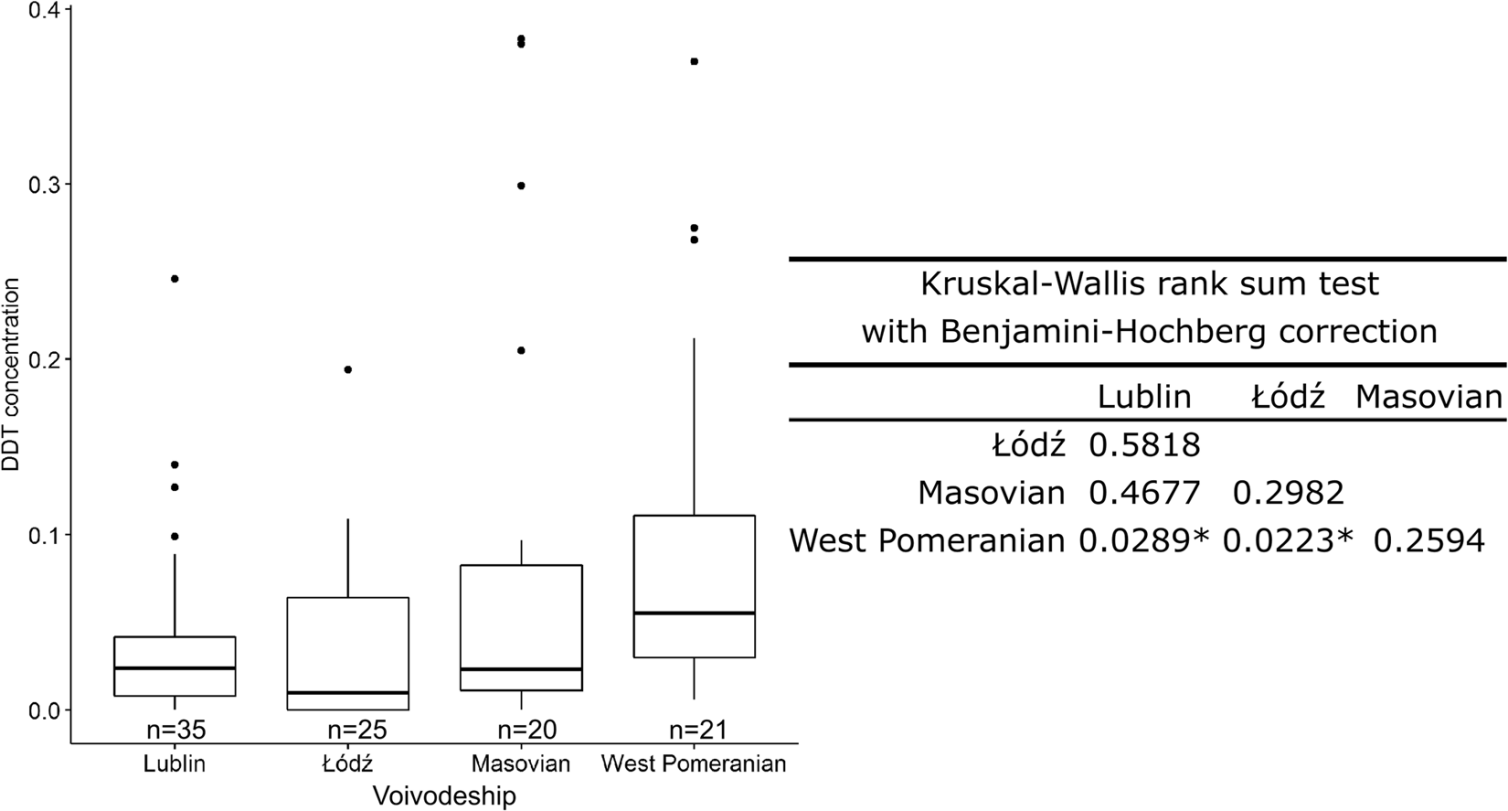
Variation of DDT content in soils of four voivodeships. The box in the figure includes the result of the Kruskal–Wallis test with Benjamini–Hochberg correction comparing the soil data from the sites of the four voivodeships. Significant (*p* < 0.05) differences are indicated by (*)

As the major part of our analysis (102 sampled fields comprising 71.83% of the monitoring data) represented four highly agricultural voivodeships characterised by contrasting soil types, crop productions and a history of farm management (Lublin, Łódź, Masovian and West Pomeranian) (see information about these factors in Table EMS2 supplemented as an Online Resource), we made an attempt to find out whether any significant correlation could be pointed out between the sampled territory and the level of contamination. The results of this analysis are presented in Fig. 2. The smallest variations in DDT contamination were observed in the Lublin voivodeship and Łódź voivodeship, which resulted to be the less contaminated ones (median = 0.010 and with the largest number of samples not containing DDT, i.e., below the detection limit, Table EMS1 supplemented as an Online Resource).

On the other hand, DDT pollution levels detected in the West Pomeranian voivodeship were significantly higher than those of the Lublin and Łódź voivodeships, falling within a comparable range of variation to those of Masovian soils. This result could be linked to the number of pesticides landfills present in those voivodeships (Witczak et al. 2013; Fig. 1a), as well as to the high incidence of large-sized, state-managed farms where DDT was frequently used at high doses (Gałuszka et al. 2011). The high level of variation of DDT contamination of Masovian soils could be explained by considering that the southern part of the voivodeship has been traditionally characterised by intensive agricultural crops, where DDT use was common—besides also being the location of several pesticide landfills (Fig. 1a). Meanwhile, the northern part is loosely cultivated and characterised by forestry and lakes, thus generally considered as little polluted. The presence of state-managed farms in the Łódź and Lublin voivodeships was far less than in the other two voivodeships (West Pomeranian and Masovia). The crops traditionally grown (cereals) and the soil texture characteristics (mainly sandy or loamy) common to both voivodeships (Niewiadomski and Tołoczko 2014; Table EMS2) are hypothesised to have also contributed for their current low contamination level. However, further research associating the soil chemical and physical characteristics to the analysis of DDT residues and, possibly, considering also the spatial variability of DDT soil residues (Li et al. 2007) would be needed to confirm these hypotheses.

### Type of DDT Contamination

The monitoring work stemmed firstly from the need of evaluating the risk of use of unauthorised pesticides in organic farms and secondly to assess the level of environmental contamination of soils managed with organic farming methods. Therefore, we have elaborated the analytical data trying to establish the possible kind of contamination (technical DDT versus formulations containing DDT impurities such as Dicofol), following the method proposed by several authors using the o,p′-DDT/p,p′-DDT ratio (Qiu et al. 2005; Qiu and Zhu 2010). Since o,p′-DDT was identified only in three highly contaminated soil samples, West Pomeranian (2) and Łódź (1) regions (Table EMS1 provided as an Online Resource), only in these cases the calculated ratio was >0, but still very low (0.21–0.33). This strongly suggests that the contamination originates from technical DDT rather than from impurities of other more recent formulations (Qiu and Zhu 2010).

**Fig. 3:**
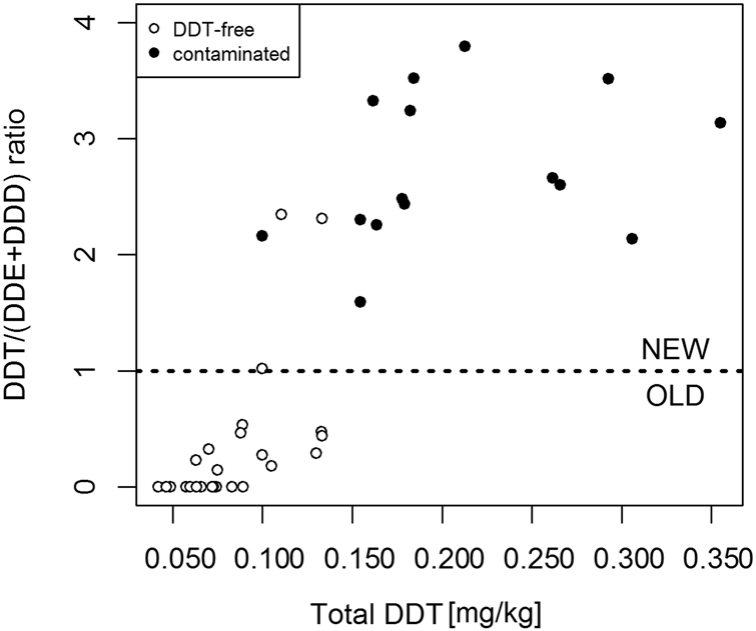
Inference of the timing of soil contamination based on DDT/(DDE + DDD) ratio in DDT-free or historically contaminated soils after addition of analytical DDT. Original DDT-free soil samples are shown in white, while previously contaminated soils are marked in black. The dotted line represents the limit value of the ratio discriminating between recent and old contamination

In the effort to better assess the period of contamination, we determined DDT residues in 40 soil samples deriving from both contaminated (*n* = 15) and uncontaminated (*n* = 25) fields which were artificially added with analytical grade p,p′-DDT. Three compounds (p,p′-DDT, p,p′-DDE and p,p′-DDD) were identified in the soil (Table 1), with an overall DDT concentration ranging from 0.042 to 0.355 mg/kg. DDT/(DDE + DDD) ratio is proposed to discriminate between old or new DDT sources in the environment (Qiu and Zhu 2010). In the present study, when classifying the samples according to this ratio, 12 out of 25 soil samples which derived from soil not found to be contaminated and artificially added with DDT should have been classified as having experienced DDT contamination in the past (Fig. 3), thus, hypothetically challenging the applicability of the proposed ratio. However, bearing in mind that these 12 samples were characterised by a relatively low ΣDDT concentration, it is considered that a p,p′-DDT concentration close to the detection limit would bias the ratio and thus the correct appraisal of the period when the contamination could have occurred. On the other hand, also some of the already contaminated soils would be incorrectly considered as being derived from uncontaminated sites. Since proper classification is a result of proportion between the historical and recent DDT contamination, an initial low DDT contamination could be considered as background contamination compared to the recent pollution. We are thus concluding that the DDT/(DDE + DDD) ratio would not be suitable to clearly distinguish the period of contamination in the case of low DDT concentration in soil (approximately up to 0.150 mg/kg)—contrary to severely polluted environments (Guo et al. 2009). Considering that the majority of our monitored fields (131 out of 142 analysed samples) fell within this low range, we were not able to adequately discriminate the sampled soils with respect to the period of their contamination.

**Table 1:** DDT and its metabolites detected in contaminated or uncontaminated soils after artificial contamination

Sample number	p,p′-DDE	p,p′-DDD	p,p′-DDT	Total DDTs concentration	DDT/(DDE + DDD) ratio	Initial purity
	Detection limit 0.005 mg/kg					
1	<LOD	0.052	<LOD	0.058	0	DDT-free
2	0.007	0.074	<LOD	0.089	0	DDT-free
3	<LOD	0.044	<LOD	0.049	0	DDT-free
4	0.007	0.060	<LOD	0.074	0	DDT-free
5	<LOD	0.038	<LOD	0.042	0	DDT-free
6	0.006	0.054	<LOD	0.066	0	DDT-free
7	0.006	0.069	<LOD	0.083	0	DDT-free
8	<LOD	0.042	<LOD	0.047	0	DDT-free
9	<LOD	0.047	0.048	0.100	1.021	DDT-free
10	<LOD	0.032	0.075	0.110	2.344	DDT-free
11	<LOD	0.039	0.090	0.133	2.308	DDT-free
12	<LOD	0.084	0.040	0.133	0.476	DDT-free
13	<LOD	0.086	0.038	0.133	0.442	DDT-free
14	0.007	0.059	<LOD	0.073	0	DDT-free
15	<LOD	0.093	0.027	0.130	0.290	DDT-free
16	<LOD	0.054	0.029	0.089	0.537	DDT-free
17	<LOD	0.047	0.011	0.063	0.234	DDT-free
18	<LOD	0.054	<LOD	0.060	0	DDT-free
19	0.006	0.054	0.009	0.075	0.146	DDT-free
20	<LOD	0.056	0.026	0.088	0.464	DDT-free
21	<LOD	0.065	<LOD	0.072	0	DDT-free
22	0.005	0.076	0.015	0.105	0.184	DDT-free
23	<LOD	0.049	0.016	0.070	0.327	DDT-free
24	0.008	0.049	<LOD	0.063	0	DDT-free
25	<LOD	0.072	0.020	0.100	0.278	DDT-free
26	0.012	0.031	0.165	0.213	3.792	Contaminated
27	0.014	0.035	0.110	0.164	2.254	Contaminated
28	0.010	0.027	0.121	0.162	3.322	Contaminated
29	0.011	0.034	0.104	0.155	2.301	Contaminated
30	0.010	0.032	0.136	0.182	3.237	Contaminated
31	0.008	0.032	0.140	0.184	3.519	Contaminated
32	0.008	0.042	0.123	0.178	2.477	Contaminated
33	0.018	0.077	0.201	0.306	2.137	Contaminated
34	0.016	0.067	0.262	0.355	3.135	Contaminated
35	0.016	0.054	0.184	0.262	2.659	Contaminated
36	0.015	0.057	0.186	0.266	2.601	Contaminated
37	0.012	0.051	0.222	0.293	3.516	Contaminated
38	0.011	0.020	0.066	0.100	2.160	Contaminated
39	0.008	0.042	0.123	0.179	2.434	Contaminated
40	0.013	0.044	0.091	0.155	1.593	Contaminated

### DDT Contamination Level in Relation to Soil pH

**Fig. 4:**
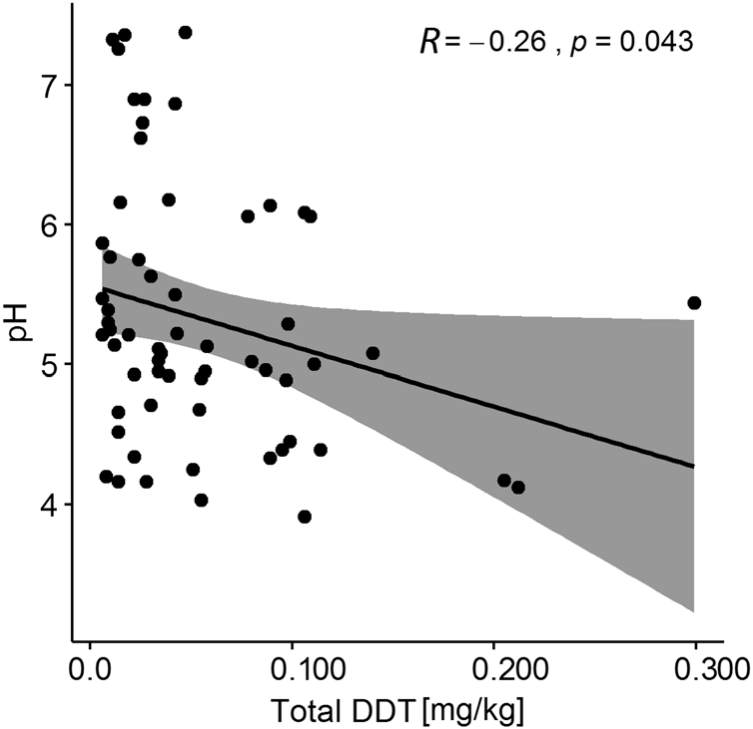
Pearson’s correlation analysis of the relationship between soil total DDT concentration and pH

To verify the possible influence of soil pH on the availability of DDT, we tested the hypothesis of finding a correlation between DDT concentration and soil pH on a subset of 61 samples among those analysed for the monitoring. These samples represented all four contamination classes defined here, for which also soil chemical analysis was available. The results are presented on Fig. 4 (detailed data in Table EMS2). The assumption we made was based on the knowledge that many microorganisms which could degrade DDT show higher degradation capability under neutral and alkaline pH (Wang et al. 2010; Pan et al. 2016), even though also the composition of the soil microbiome could impact on the degradation capacity of DDT both directly (Regar et al. 2019; Gaur et al. 2018) or indirectly, e.g., through the action of earthworms (Xu et al. 2019). However, the Pearson correlation coefficient (*R* = −0.26, *p* = 0.043) indicated a weak negative correlation between DDT concentration and pH (Fig. 4), with only few soil samples having high DDT content deriving from soils with a low pH value. Even though a possible explanation of this result could derive from the limited number of samples assessed, it is known that several other environmental factors (i.e., soil texture, application history, climatic conditions, etc.), some of which are presented in Table EMS2, can affect the persistence or degradation of DDT residues (Samuel and Pillai 1989; Boul et al. 1994; Xu et al. 1994, p. 199; Zayed et al. 1994; Spencer et al. 1996; Zhao et al. 2010), thus modifying their bioavailability. The differences between the sampled fields in relation to these factors could thus account for the limited correlation found. Furthermore, basic soil conditions have been reported to enhance the transformation of p,p′-DDT into p,p′-DDE (Nash et al. 1973). Nevertheless, no relationship was found between soil pH and organochloride pesticides residues in the nation-wide monitoring of Polish soils (Ukalska-Jaruga et al. 2020).

A similar lack of correlation resulted also when DDT content was compared to the amount of organic matter in the soil sample (data not shown). Humic acids can increase the apparent solubility of DDT (Carter and Suffet 1982), but amending the soil with high quantities of organic matter was found to significantly retard DDT biodegradation, possibly due to binding of DDT to dissolved organic carbon (Kantachote et al. 2004). Further research on the complex interactions between soil physical–chemical properties, its microbiome, including the microbial functional degradation potential, and the crops’ physiological contribution to the soil environment in relation to the possibilities to degrade DDT is thus needed to understand the behaviour of DDT residues (Sun et al. 2015), particularly under climate change conditions (Gaur et al. 2018). However, even though seasonal variation in DDT concentrations has been reported in soils (Zhang et al. 2013), in agreement with the finding of differential release of residues into atmosphere (Motelay-Massei et al. 2005; Liu et al. 2009), sampling consistently at the end of summer during our monitoring exercise should have also reduced the risk of a bias in the results due to seasonal variability.

### Uptake of DDT Residues by Plants

Another important issue associated to the monitoring of pesticide presence in the agro-eco system is the translocation of these hazardous compounds from the soil to the edible parts of crops. To investigate this aspect, we analysed DDT residue content in various crops cultivated on a subset of the monitored fields (30) representing all four soil contamination categories (below the detection level, low, medium and high DDT contamination)—the results are presented in Table 2. In five cases, due to the limited area of the horticultural crop growing on the field (which is typical for organic farms), several plant species were gathered from a single field. Most of the analyses concerned the edible part of the plant, but in fourteen cases also other plant tissues were analysed. The majority of plant samples (61 out of 67, i.e., 91%) did not contain detectable DDT residues, regardless of the initial soil contamination level. The ΣDDT concentration of the six samples where residues were determined never exceeded 0.012 mg/kg, thus well below the EU maximum residue level. The residues were found in root tissues, including edible parts, and only in one case (raspberry) they were detected in leaves (not edible), but not in the fruits. Three of these cases were found in roots of vegetable plants (cabbage, leek and celeriac) growing on soil where DDT was not detected. On the other hand, the other three contaminated plant samples were collected from soils classified as medium contaminated (Table 2). These results have not allowed to observe any trend showing a possible association between the soil contamination level and the DDT content of the analysed crops.

**Table 2:** DDT residues in different crops grown on monitored sites. Data are sorted according to the contamination level of the soil samples

Location/plant organ	DDT and its metabolites content						Total DDT content
	o,p-DDE	p,p-DDE	p,p-DDD	p,p-DDT	o,p-DDT	o,p-DDD	
	Detection limit 0.005 mg/kg						
**Uncontaminated soils**							
Brzostówka 1	<LOD	<LOD	<LOD	<LOD	<LOD	<LOD	<LOD
Cabbage, shoots and leaves	<LOD	<LOD	<LOD	<LOD	<LOD	<LOD	<LOD
Cabbage, roots	<LOD	0.005	<LOD	<LOD	<LOD	<LOD	0.006
Carrot, shoots and leaves	<LOD	<LOD	<LOD	<LOD	<LOD	<LOD	<LOD
Carrot, roots	<LOD	<LOD	<LOD	<LOD	<LOD	<LOD	<LOD
Parsley, shoots and leaves	<LOD	<LOD	<LOD	<LOD	<LOD	<LOD	<LOD
Parsley, roots	<LOD	<LOD	<LOD	<LOD	<LOD	<LOD	<LOD
Parsnip, shoots and leaves	<LOD	<LOD	<LOD	<LOD	<LOD	<LOD	<LOD
Parsnip, roots	<LOD	<LOD	<LOD	<LOD	<LOD	<LOD	<LOD
Celery, shoots and leaves	<LOD	<LOD	<LOD	<LOD	<LOD	<LOD	<LOD
Celery, tuber	<LOD	<LOD	<LOD	<LOD	<LOD	<LOD	<LOD
Celery, roots	<LOD	0.023	<LOD	0.008	<LOD	<LOD	0.034
Fennel, shoots and leaves	<LOD	<LOD	<LOD	<LOD	<LOD	<LOD	<LOD
Fennel, tuber	<LOD	<LOD	<LOD	<LOD	<LOD	<LOD	<LOD
Fennel, roots	<LOD	<LOD	<LOD	<LOD	<LOD	<LOD	<LOD
Leek, leaves	<LOD	<LOD	<LOD	<LOD	<LOD	<LOD	<LOD
Leek, roots	<LOD	0.006	<LOD	<LOD	<LOD	<LOD	0.007
Red onion	<LOD	<LOD	<LOD	<LOD	<LOD	<LOD	<LOD
Onion	<LOD	<LOD	<LOD	<LOD	<LOD	<LOD	<LOD
Dąbrowiec	<LOD	<LOD	<LOD	<LOD	<LOD	<LOD	<LOD
Lentil	<LOD	<LOD	<LOD	<LOD	<LOD	<LOD	<LOD
Jastrzębna 2	<LOD	<LOD	<LOD	<LOD	<LOD	<LOD	<LOD
Cereals mix	<LOD	<LOD	<LOD	<LOD	<LOD	<LOD	<LOD
Godzianów 1	<LOD	<LOD	<LOD	<LOD	<LOD	<LOD	<LOD
Buckwheat	<LOD	<LOD	<LOD	<LOD	<LOD	<LOD	<LOD
Godzianów 2	<LOD	<LOD	<LOD	<LOD	<LOD	<LOD	<LOD
Rapeseed	<LOD	<LOD	<LOD	<LOD	<LOD	<LOD	<LOD
Godzianów 3	<LOD	<LOD	<LOD	<LOD	<LOD	<LOD	<LOD
Triticale	<LOD	<LOD	<LOD	<LOD	<LOD	<LOD	<LOD
Godzianów 4	<LOD	<LOD	<LOD	<LOD	<LOD	<LOD	<LOD
Phacelia	<LOD	<LOD	<LOD	<LOD	<LOD	<LOD	<LOD
Godzianów 5	<LOD	<LOD	<LOD	<LOD	<LOD	<LOD	<LOD
Synapis	<LOD	<LOD	<LOD	<LOD	<LOD	<LOD	<LOD
Suliszew	<LOD	<LOD	<LOD	<LOD	<LOD	<LOD	<LOD
Potato, tuber	<LOD	<LOD	<LOD	<LOD	<LOD	<LOD	<LOD
Wysokienice	<LOD	<LOD	<LOD	<LOD	<LOD	<LOD	<LOD
Pumpkin	<LOD	<LOD	<LOD	<LOD	<LOD	<LOD	<LOD
**Low contaminated soils**							
Brzostówka 5	<LOD	0.005	<LOD	<LOD	<LOD	<LOD	0.006
Pumpkin	<LOD	<LOD	<LOD	<LOD	<LOD	<LOD	<LOD
Jastrzębna 1	<LOD	0.006	<LOD	<LOD	<LOD	<LOD	0.007
Potato	<LOD	<LOD	<LOD	<LOD	<LOD	<LOD	<LOD
Zębowo	<LOD	0.007	<LOD	<LOD	<LOD	<LOD	0.007
Potato	<LOD	<LOD	<LOD	<LOD	<LOD	<LOD	<LOD
Dębowa Góra 1	<LOD	<LOD	<LOD	0.008	<LOD	<LOD	0.008
Cherry	<LOD	<LOD	<LOD	<LOD	<LOD	<LOD	<LOD
Skierniewice 2	<LOD	0.005	<LOD	0.007	<LOD	<LOD	0.013
Tomato, fruits	<LOD	<LOD	<LOD	<LOD	<LOD	<LOD	<LOD
Tomato, leaves	<LOD	<LOD	<LOD	<LOD	<LOD	<LOD	<LOD
Tomato, roots	<LOD	<LOD	<LOD	<LOD	<LOD	<LOD	<LOD
Kamion 1	<LOD	0.017	<LOD	<LOD	<LOD	<LOD	0.019
Potato, tuber	<LOD	<LOD	<LOD	<LOD	<LOD	<LOD	<LOD
**Medium contaminated soils**							
Turowo	<LOD	0.016	0.010	<LOD	<LOD	<LOD	0.029
Common thyme	<LOD	<LOD	<LOD	<LOD	<LOD	<LOD	<LOD
Oenothera	<LOD	<LOD	<LOD	<LOD	<LOD	<LOD	<LOD
Cetyń	<LOD	0.017	<LOD	0.021	<LOD	<LOD	0.040
Potato, tuber	<LOD	<LOD	<LOD	<LOD	<LOD	<LOD	<LOD
Tchórzenica	<LOD	0.015	0.0095	0.022	<LOD	<LOD	0.049
Black currant, fruits	<LOD	<LOD	<LOD	<LOD	<LOD	<LOD	<LOD
Black currant, leaves	<LOD	<LOD	<LOD	<LOD	<LOD	<LOD	<LOD
Skierniewice 1	<LOD	0.025	0.008	0.020	<LOD	<LOD	0.057
Potato	<LOD	<LOD	<LOD	<LOD	<LOD	<LOD	<LOD
Zucchini	<LOD	<LOD	<LOD	<LOD	<LOD	<LOD	<LOD
Skierniewice 3	<LOD	0.028	0.014	0.011	<LOD	<LOD	0.058
Common bean	<LOD	<LOD	<LOD	<LOD	<LOD	<LOD	<LOD
Beetroot	<LOD	<LOD	<LOD	<LOD	<LOD	<LOD	<LOD
Onion	<LOD	<LOD	<LOD	<LOD	<LOD	<LOD	<LOD
Carrot	<LOD	<LOD	<LOD	<LOD	<LOD	<LOD	<LOD
Cucumber	<LOD	<LOD	<LOD	<LOD	<LOD	<LOD	<LOD
Potato	<LOD	<LOD	<LOD	<LOD	<LOD	<LOD	<LOD
Zucchini	<LOD	<LOD	<LOD	<LOD	<LOD	<LOD	<LOD
Rumex	<LOD	<LOD	<LOD	<LOD	<LOD	<LOD	<LOD
Rheum	<LOD	<LOD	<LOD	<LOD	<LOD	<LOD	<LOD
Tomato	<LOD	<LOD	<LOD	<LOD	<LOD	<LOD	<LOD
Dill	<LOD	<LOD	<LOD	<LOD	<LOD	<LOD	<LOD
Tomato, fruits	<LOD	<LOD	<LOD	<LOD	<LOD	<LOD	<LOD
Tomato, leaves	<LOD	<LOD	<LOD	<LOD	<LOD	<LOD	<LOD
Tomato, roots	<LOD	0.010	<LOD	<LOD	<LOD	<LOD	0.011
Żabówko	<LOD	0.029	0.023	<LOD	<LOD	0.006	0.064
Apple tree, fruits	<LOD	<LOD	<LOD	<LOD	<LOD	<LOD	<LOD
Apple tree, leaves	<LOD	<LOD	<LOD	<LOD	<LOD	<LOD	<LOD
Kamion 2	<LOD	0.028	0.008	0.025	<LOD	<LOD	0.065
Potato, tuber	<LOD	<LOD	<LOD	<LOD	<LOD	<LOD	<LOD
Radziejów	<LOD	0.040	0.021	<LOD	<LOD	<LOD	0.068
Corn	<LOD	<LOD	<LOD	<LOD	<LOD	<LOD	<LOD
Goszyce	<LOD	0.038	0.007	0.039	<LOD	<LOD	0.089
Raspberry, leaves	<LOD	0.010	<LOD	<LOD	<LOD	<LOD	0.012
Rossocha	<LOD	0.016	0.018	0.061	<LOD	<LOD	0.099
Carrot	<LOD	0.009	<LOD	<LOD	<LOD	<LOD	0.010
**High contaminated soils**							
Sycewice	<LOD	0.100	0.044	<LOD	<LOD	<LOD	0.160
Potato, tuber	<LOD	<LOD	<LOD	<LOD	<LOD	<LOD	<LOD
Dębowa Góra 2	<LOD	0.037	0.025	0.126	<LOD	<LOD	0.194
Currant, leaves	<LOD	<LOD	<LOD	<LOD	<LOD	<LOD	<LOD
Stepniczka	<LOD	0.072	0.139	<LOD	<LOD	0.031	0.268
Apple tree, fruits	<LOD	<LOD	<LOD	<LOD	<LOD	<LOD	<LOD
Gean, leaves	<LOD	<LOD	<LOD	<LOD	<LOD	<LOD	<LOD
Krukowo	<LOD	0.300	0.031	<LOD	<LOD	0.013	0.383
Fruit trees, leaves	<LOD	<LOD	<LOD	<LOD	<LOD	<LOD	<LOD

Differences in the capacity of diverse crops and species to uptake DDT or other organochlorine pesticides residues have been confirmed (White and Zeeb 2007; Mo et al. 2008). These results are further supported by the data presented as well as by other studies that we have carried out on different species (about a dozen) and more than forty genotypes of *C. pepo* (unpublished data). The presence of DDT residues in plants growing on soils from which samples were not found to be contaminated is further pointing out the issue of spatial variation of soil DDT contamination (Zhang et al. 2013). This aspect is of utmost importance when the analytical result can lead to the refusal of produce certification, as the case of farms operating within the organic farming or other certification systems. Recent cases that emerged in Europe (EFSA European Food Safety Authority 2018c) have fuelled the discussion about the functioning of the control system and the protection of consumers. This particular aspect has prompted the launch of a specific monitoring programme at EU level, with reassuring results (EFSA European Food Safety Authority 2018c). The capacity of plants and their root-associated microbiome, particularly arbuscular mycorrhiza and plant growth promoting rhizobacteria, to mobilise strongly soil-bound DDT (White 2001; Wu et al. 2008; Lenoir et al. 2016; Rani et al. 2019) could, however, also account for the detection of the residues in plants collected from soils where DDT was not analytically detected.

## Conclusions

In this study, we presented data of DDT residues in Polish agricultural soils managed according to organic farming rules, which are showing that contamination from this persistent organic pesticide is still posing a threat for detection of residues in crops, as evidenced in several agricultural areas in the world. Samples from more than 80% of examined sites contained detectable amounts of DDT or its metabolites. The distribution of these residues appears to reflect to a broader extent the historical agricultural use of the sampled soils and, possibly, also the vicinity of underground landfills of hazardous compounds. However, the occurrence of DDT residues was found to be lower in comparison to a similar monitoring carried out in Poland on arable fields managed by conventional methods. Furthermore, from the analysis of plants grown on the sampled soils, it appeared that almost all crops resulted not to be contaminated from DDT residues. The few cases of pesticide residues detection were mainly from root tissues, which would allow considering unlikely the use of unauthorised compounds. This result is pointing to the need of rigorous controls at farm level, particularly for quality production methods such as organic farming, especially during the conversion period to reduce the risk of having fields contaminated with DDT entering the quality production scheme. Such result was found to be in line with the outcomes of an analysis using EU-wide monitoring data on organic food (EFSA European Food Safety Authority 2018a), further supporting the conclusions that the risk of contamination also for organic products is likely derived from general environment pollution levels rather than from the use of unauthorised substances in organic farms. The safety of organic products should thus not be expected to be lower than conventional food in this respect, but possibly higher due to the positive effect of this cultivation system on soil biological fertility. The use of the DDT/(DDE + DDD) ratio to appraise the source or period of contamination pointed out that its usage in the case of a low level of soil contamination could be less reliable than in highly contaminated soils.

## Supplementary information

Supplementary Table S1

Supplementary Table S2

## References

[CR1] Arias AH, Pereyra MT, Marcovecchio JE (2011). Multi-year monitoring of estuarine sediments as ultimate sink for DDT, HCH, and other organochlorinated pesticides in Argentina. Environ Monit Assess.

[CR2] Bański J (2011). Changes in agricultural land ownership in Poland in the period of the market economy. Agric Econ.

[CR3] Boller EF, Van Lenteren JC, Delucci V (2006). IOBC—history of the first 50 years (1956–2006).

[CR4] Borowik A, Wyszkowska J, Kucharski J (2017). Response of microorganisms and enzymes to soil contamination with a mixture of terbuthylazine, mesotrione, and S-metolachlor. Environ Sci Pollut Res.

[CR5] Boul HL, Garnham ML, Hucker D (1994). Influence of agricultural practices on the levels of DDT and its residues in soil. Environ Sci Technol.

[CR6] Carter CW, Suffet IH (1982). Binding of DDT to dissolved humic materials. Environ Sci Technol.

[CR7] Commission Regulation (EC) (2008) Commission Regulation (EC) No 889/2008 of 5 September 2008 laying down detailed rules for the implementation of Council Regulation (EC) No 834/2007 on organic production and labelling of organic products with regard to organic production, labelling and control. Off Journal L 250:1–84

[CR8] EFSA European Food Safety Authority (2018a) Monitoring data on pesticide residues in food: results on organic versus conventionally produced food. EFSA Supporting Publication 2018:EN-1397, Parma, Italy. 10.2903/sp.efsa.2018.en-1397

[CR9] EFSA European Food Safety Authority (2018b) National summary reports on pesticide residue analysis performed in 2016. EFSA Supporting Publication 2018:EN-1454, Parma, Italy. 10.2903/sp.efsa.2018.EN-1454

[CR10] EFSA European Food Safety Authority (2018c) The 2016 European Union report on pesticide residues in food. EFSA J. 16:5348. 10.2903/j.efsa.2018.5348 10.2903/j.efsa.2018.5348 PMC7009629 32625983

[CR11] Gałuszka A, Migaszewski ZM, Manecki P (2011). Pesticide burial grounds in Poland: a review. Environ Int.

[CR12] Gaur N, Narasimhulu K, PydiSetty Y (2018). Recent advances in the bio-remediation of persistent organic pollutants and its effect on environment. J Clean Production.

[CR13] GIOŚ Main Inspectorate of Environmental Protection (2015) The monitoring of chemism of arable soils in Poland. GIOŚ Main Inspectorate of Environmental Protection, Warsaw, Poland

[CR14] Gorlach E, Mazur T (2002) Chemia rolna. PWN Warsaw, Poland

[CR15] Guo Y, Yu HY, Zeng EY (2009). Occurrence, source diagnosis, and biological effect assessment of DDT and its metabolites in various environmental compartments of the Pearl River Delta, South China: a review. Environ Pollut.

[CR16] Hung H, Katsoyiannis AA, Guardans R (2016). Ten years of global monitoring under the Stockholm Convention on Persistent Organic Pollutants (POPs): trends, sources and transport modelling. Environ Pollut.

[CR17] Ignatowicz K (2009). Badania rozpoznawcze mozliwosci zastosowania fitoremediacji do ochrony terenow wokół mogilników pestycydowych. Rocznik Ochrony Srodowiska.

[CR18] Iwata H, Tanabe S, Sakal N, Tatsukawa R (1993). Distribution of persistent organochiorines in the oceanic air and surface seawater and the role of ocean on their global transport and fate. Environ Sci Technol.

[CR19] Jiao S, Chen W, Wang E (2016). Microbial succession in response to pollutants in batch-enrichment culture. Sci Rep.

[CR20] Kantachote D, Naidu R, Williams B (2004). Bioremediation of DDT-contaminated soil: enhancement by seaweed addition. J Chem Technol Biotechnol.

[CR21] Lenoir I, Lounes-Hadj Sahraoui A, Fontaine J (2016). Arbuscular mycorrhizal fungal-assisted phytoremediation of soil contaminated with persistent organic pollutants: a review. Eur J Soil Sci.

[CR22] Lewis KA, Tzilivakis J, Warner DJ, Green A (2016). An international database for pesticide risk assessments and management. Hum Ecol Risk Assess.

[CR23] Li BG, Ran Y, Cao J, Liu WX, Shen WR, Wang XJ, Coveney RM, Tao S (2007). Spatial structure analysis and kriging of dichlorodiphenyltrichloroethane residues in topsoil from Tianjin, China. Geoderma.

[CR24] Li H, Wang J, Liu Q, Zhou Z, Chen F, Xiang D (2019). Effects of consecutive monoculture of sweet potato on soil bacterial community as determined by pyrosequencing. J Basic Microbiol.

[CR25] Liu X, Zhang G, Li G (2009). Seasonal patterns and current sources of DDTs, Chlordanes, Hexachlorobenzene, and Endosulfan in the atmosphere of 37 Chinese cities. Environ Sci Technol.

[CR26] Maeder P, Fliessbach A, Dubois D (2002). Soil fertility and biodiversity in organic farming. Science.

[CR27] Manz M, Wenzel K-D, Dietze U, Schüürmann G (2001). Persistent organic pollutants in agricultural soils of central Germany. Sci Total Environ.

[CR28] Matyjaszczyk E (2018). Plant protection means used in organic farming throughout the European Union. Pest Manag Sci.

[CR29] Mo C-H, Cai Q-Y, Li H-Q (2008). Potential of different species for use in removal of DDT from the contaminated soils. Chemosphere.

[CR30] Motelay-Massei A, Harner T, Shoeib M, Diamond M, Stern G, Rosenberg B (2005). Using passive air samplers to assess urban–rural trends for persistent organic pollutants and polycyclic aromatic hydrocarbons: 2. Seasonal trends for PAHs, PCBs, and organochlorine pesticides. Environ Sci Technol.

[CR31] Nash RG, Harris WG, Lewis CC (1973). Soil pH and metallic amendment effects on DDT conversion to DDE. J Environ Qual.

[CR32] Niewiadomska A, Zmudzki J (2011) Chlorinated hydrocarbons in animal tissues and producs of animal origin from Poland. In: Loganathan BG, Lam PK-S (eds) Global contamination trends of persistent organic chemicals. CRC Press, Boca Raton, FL, USA, p 337–353

[CR33] Niewiadomski A, Tołoczko W (2014) Characteristics of soil cover in Poland with special attention paid to the Łódź region. In: Kobojek E, Marszał T (eds) Natural environment of Poland and its protection in Łódź University geographical research. Łódź University Press, Łódź

[CR34] Pan X, Lin D, Zheng Y (2016). Biodegradation of DDT by *Stenotrophomonas* sp. DDT-1: characterization and genome functional analysis. Sci Rep.

[CR35] Polish Official Gazette (2013) Regulation of the Minister of Agriculture and Rural Development from 27 November 2013 on the sampling of plants, plant products or other objects to test for the presence of residues of plant protection products. Dz.U. 2013 poz. 1549

[CR36] Polish Official Gazette (2016) Regulation of the Minister of Environment from 01 September 2016 on the standards of evaluation of the surface soil contamination. Dz.U. 2016 poz. 1395

[CR37] Plaza-Bolaños P, Padilla-Sánchez JA, Garrido-Frenich A (2012). Evaluation of soil contamination in intensive agricultural areas by pesticides and organic pollutants: South-eastern Spain as a case study. J Environ Monit.

[CR38] Qiu X, Zhu T (2010). Using the o,p’-DDT/p,p’-DDT ratio to identify DDT sources in China. Chemosphere.

[CR39] Qiu X, Zhu T, Yao B (2005). Contribution of dicofol to the current DDT pollution in China. Environ Sci Technol.

[CR40] Qu C, Albanese S, Chen W (2016). The status of organochlorine pesticide contamination in the soils of the Campanian Plain, southern Italy, and correlations with soil properties and cancer risk. Environ Pollut.

[CR41] R Core Team (2019). R: a Language and Environment for Statistical Computing.

[CR42] Rani R, Kumar V, Gupta P and Chandra A (2019) Application of plant growth promoting rhizobacteria in remediation of pesticides contaminated stressed soil. In: Singh JS (ed.) New and future developments in microbial biotechnology and bioengineering. Elsevier B.V., Amsterdam, The Netherland, p 341–353. 10.1016/B978-0-12-818258-1.00029-7

[CR43] Regar RK, Gaur VK, Bajaj A (2019). Comparative microbiome analysis of two different long-term pesticide contaminated soils revealed the anthropogenic influence on functional potential of microbial communities. Sci Total Environ.

[CR44] Růžičková P, Klánová J, Čupr P (2008). An assessment of air-soil exchange of polychlorinated biphenyls and organochlorine pesticides across central and Southern Europe. Environ Sci Technol.

[CR45] Samuel T, Pillai MKK (1989). The effect of temperature and solar radiations on volatilisation, mineralisation and degradation of [14C]-DDT in soil. Environ Pollut.

[CR46] Silva V, Mol HGJ, Zomer P (2019). Pesticide residues in European agricultural soils—a hidden reality unfolded. Sci Total Environ.

[CR47] Siłowiecki A (2002) Inwentaryzacja odpadów środków ochrony roślin (Inventory of plant protection products waste). Global Environment Project in Poland. GF/POL/INV/R.10. Warsaw, Poland. p 45

[CR48] Škrbić B, Durišić-Mladenović N (2007). Distribution of chlorinated organic pollutants in a wide variety of soils from Europe and Asia: a multivariate statistical approach. Arch Environ Contam Toxicol.

[CR49] Spencer WF, Singh G, Taylor CD (1996). DDT persistence and volatility as affected by management practices after 23 years. J Environ Qual.

[CR50] Sun G, Zhang X, Hu Q, Zhang H, Zhang D, Li G (2015). Biodegradation of dichlorodiphenyltrichloroethanes (DDTs) and hexachlorocyclohexanes (HCHs) with plant and nutrients and their effects on the microbial ecological kinetics. Microb Ecol.

[CR51] Tarcau D, Cucu-Man S, Boruvkova J (2013). Organochlorine pesticides in soil, moss and tree-bark from North-Eastern Romania. Sci Total Environ.

[CR52] Tartanus M, Malusá E, Labanowska BH (2017). DDT content in polish soils—current state and attempts of rhizo-bioremediation. J Res Appl Agric Eng.

[CR53] Thiombane M, Petrik A, Di Bonito M (2018). Status, sources and contamination levels of organochlorine pesticide residues in urban and agricultural areas: a preliminary review in central–southern Italian soils. Environ Sci Pollut Res.

[CR54] Tieyu W, Yonglong L, Hong Z, Yajuan S (2005). Contamination of persistent organic pollutants (POPs) and relevant management in China. Environ Int.

[CR55] Ukalska-Jaruga A, zena SmreczakB, Siebielec G (2020). Assessment of pesticide residue content in Polish agricultural soils. Molecules.

[CR56] Villanneau EJ, Saby NPA, Marchant BP (2011). Which persistent organic pollutants can we map in soil using a large spacing systematic soil monitoring design? A case study in Northern France. Sci Total Environ.

[CR57] Wang G, Zhang J, Wang L (2010). Co-metabolism of DDT by the newly isolated bacterium, Pseudoxanthomonas sp. wax. Braz J Microbiol.

[CR58] White JC (2001). Plant-facilitated mobilization and translocation of weathered 2,2-bis(p-chlorophenyl)-1,1-dichloroethylene (p,p′-DDE) from an agricultural soil. Environ Toxicol Chem.

[CR59] White JC, Zeeb BA (2007) Plant phylogeny and the remediation of persistent organic pollutants. In: Willey N (ed) Phytoremediation: methods and reviews. Humana Press, Totowa, NJ, p 71–87

[CR60] Witczak A, Mituniewicz-Małek A, Dmytrów I (2013). Assessment of daily intake of organochlorine pesticides from milk in different regions of Poland. J Environ Sci Health Part B.

[CR61] Wu N, Zhang S, Huang H (2008). DDT uptake by arbuscular mycorrhizal alfalfa and depletion in soil as influenced by soil application of a non-ionic surfactant. Environ Pollut.

[CR62] Xu H-J, Bai J, Li W-Y, Zhao L-X, Li Y-T (2019). Removal of persistent DDT residues from soils by earthworms: a mechanistic study. J Hazard Mater.

[CR63] Xu B, Jianying G, Yongxi Z, Haibo L (1994). Behaviour of ddt in chinese tropical soils. J Environ Sci Health Part B.

[CR64] Zayed SMAD, Mostafa IY, El-Arab AE (1994). Degradation and fate of 14C-DDT and 14C-DDE in Egyptian soil. J Environ Sci Health Part B.

[CR65] Zhang F, He J, Yao Y (2013). Spatial and seasonal variations of pesticide contamination in agricultural soils and crops sample from an intensive horticulture area of Hohhot, North-West China. Environ Monit Assess.

[CR66] Zhao Y, Yi X, Li M (2010). Biodegradation kinetics of DDT in soil under different environmental conditions by laccase extract from white rot fungi. Chin J Chem Eng.

