# Supplementary material for: Monitoring of DDT in Agricultural Soils under Organic Farming in Poland and the Risk of Crop Contamination
Source: Environ Manage. 2020 Aug 19;66(5):916–29. doi: 10.1007/s00267-020-01347-9 (PMC7591450; doi:10.1007/s00267-020-01347-9)
Supplement: Supplementary file 1 — Supplementary Table S1 [file 267_2020_1347_MOESM1_ESM.pdf]

Table EMS1. The content of DDT and its metabolites in soil samples collected from field managed according to organic farming rules in different locations of Poland.

Sample number	Voivodeship	Localization	o,p'-DDE	p,p'-DDE	p,p'-DDD	p,p'-DDT	o,p'-DDT	o,p'-DDD	∑DDT concentration	Contamination category
			detection limit 0.005 mg/kg							
1	Greater Poland	Bednary	<LOD	<LOD	<LOD	0.007	<LOD	<LOD	0.007	low
2	Greater Poland	Lubień1	<LOD	0.006	<LOD	0.006	<LOD	<LOD	0.012	low
3	Greater Poland	Lubień2	<LOD	0.010	<LOD	0.011	<LOD	<LOD	0.022	low
4	Greater Poland	NowyDwór	<LOD	0.024	0.015	<LOD	<LOD	<LOD	0.043	medium
5	Greater Poland	NowyDwór1	<LOD	0.029	0.010	<LOD	<LOD	<LOD	0.043	medium
6	Greater Poland	NowyDwór2	<LOD	0.032	<LOD	0.020	<LOD	<LOD	0.055	medium
7	Greater Poland	Skarszew	<LOD	<LOD	<LOD	0.011	<LOD	<LOD	0.011	low
8	Greater Poland	Wiry	<LOD	0.028	<LOD	0.011	<LOD	<LOD	0.042	medium
9	Greater Poland	Zawada	<LOD	<LOD	<LOD	<LOD	<LOD	<LOD	<LOD	no
10	Greater Poland	Zębowo	<LOD	0.007	<LOD	<LOD	<LOD	<LOD	0.007	low
11	Kuyavian-Pomeranian	Radziejów	<LOD	0.040	0.021	<LOD	<LOD	<LOD	0.068	medium
12	Kuyavian-Pomeranian	Turowo	<LOD	0.016	0.010	<LOD	<LOD	<LOD	0.029	medium
13	Lesser Poland	Brzezna1	<LOD	<LOD	<LOD	<LOD	<LOD	<LOD	<LOD	no
14	Lesser Poland	Brzezna2	<LOD	<LOD	<LOD	<LOD	<LOD	<LOD	<LOD	no
15	Lesser Poland	Goszyce	<LOD	0.038	0.007	0.039	<LOD	<LOD	0.089	medium
16	Lesser Poland	Maszkowice1	<LOD	0.008	<LOD	<LOD	<LOD	<LOD	0.009	low
17	Lesser Poland	Maszkowice2	<LOD	0.008	<LOD	0.007	<LOD	<LOD	0.015	low
18	Lower Silesian	Biestrzyków	<LOD	0.080	<LOD	0.017	<LOD	<LOD	0.106	medium
19	Lower Silesian	Chocieszów1	<LOD	0.033	<LOD	0.021	<LOD	<LOD	0.058	medium
20	Lower Silesian	Chocieszów2	<LOD	0.021	<LOD	0.011	<LOD	<LOD	0.034	medium
21	Lower Silesian	Lipiany1	<LOD	0.019	<LOD	0.009	<LOD	<LOD	0.030	medium
22	Lower Silesian	Lipiany2	<LOD	0.025	<LOD	0.014	<LOD	<LOD	0.042	medium
23	Lublin	Andrzejów	<LOD	0.048	0.010	0.025	<LOD	<LOD	0.089	medium
24	Lublin	Borki	<LOD	0.021	<LOD	0.020	<LOD	<LOD	0.044	medium
25	Lublin	Brzostówka1	<LOD	<LOD	<LOD	<LOD	<LOD	<LOD	<LOD	no
26	Lublin	Brzostówka2	<LOD	0.011	0.005	0.007	<LOD	<LOD	0.024	low
27	Lublin	Brzostówka3	<LOD	0.012	0.006	0.012	<LOD	<LOD	0.031	medium
28	Lublin	Brzostówka4	<LOD	<LOD	<LOD	<LOD	<LOD	<LOD	<LOD	no
29	Lublin	Brzostówka5	<LOD	0.005	<LOD	<LOD	<LOD	<LOD	0.006	low

Sample number	Voivodeship	Localization	o,p'-DDE	p,p'-DDE	p,p'-DDD	p,p'-DDT	o,p'-DDT	o,p'-DDD	∑DDT concentration	Contamination category
			detection limit 0.005 mg/kg							
30	Lublin	GóryKluczkowickie	<LOD	0.046	0.01	0.025	<LOD	<LOD	0.087	medium
31	Lublin	GrabówRycki1	<LOD	0.010	<LOD	0.013	<LOD	<LOD	0.025	medium
32	Lublin	GrabówRycki2	<LOD	0.010	<LOD	0.015	<LOD	<LOD	0.026	medium
33	Lublin	GrabówRycki3	<LOD	0.027	<LOD	0.025	<LOD	<LOD	0.055	medium
34	Lublin	GrabówRycki4	<LOD	0.013	<LOD	0.025	<LOD	<LOD	0.039	medium
35	Lublin	Hulcze	<LOD	<LOD	<LOD	<LOD	<LOD	<LOD	<LOD	no
36	Lublin	Jastków	<LOD	0.011	<LOD	0.012	<LOD	<LOD	0.024	low
37	Lublin	KarczmiskaDrugie1	<LOD	0.015	<LOD	0.019	<LOD	<LOD	0.035	medium
38	Lublin	KarczmiskaDrugie2	<LOD	0.009	<LOD	<LOD	<LOD	<LOD	0.010	low
39	Lublin	Krzewica1	<LOD	0.010	<LOD	<LOD	<LOD	<LOD	0.011	low
40	Lublin	Krzewica2	<LOD	0.012	<LOD	<LOD	<LOD	<LOD	0.014	low
41	Lublin	Leopoldów	<LOD	<LOD	<LOD	0.005	<LOD	<LOD	0.005	low
42	Lublin	Łopatki	<LOD	0.042	0.0064	0.052	0.013	0.007	0.127	high
43	Lublin	PiaskiSzlacheckie1	<LOD	0.005	<LOD	0.006	<LOD	<LOD	0.012	low
44	Lublin	PiaskiSzlacheckie2	<LOD	0.006	<LOD	0.007	<LOD	<LOD	0.015	low
45	Lublin	PisakiSzlacheckie3	<LOD	<LOD	<LOD	0.010	<LOD	<LOD	0.010	low
46	Lublin	Przestrzeń1	<LOD	0.017	<LOD	0.020	<LOD	<LOD	0.039	medium
47	Lublin	Przestrzeń2	<LOD	0.012	<LOD	0.010	<LOD	<LOD	0.022	low
48	Lublin	Przestrzeń3	<LOD	<LOD	<LOD	<LOD	<LOD	<LOD	<LOD	no
49	Lublin	Wincentów	<LOD	<LOD	<LOD	<LOD	<LOD	<LOD	<LOD	no
50	Lublin	WolaSkromowska1	<LOD	<LOD	<LOD	<LOD	<LOD	<LOD	<LOD	no
51	Lublin	WolaSkromowska2	<LOD	0.006	<LOD	0.012	<LOD	<LOD	0.018	low
52	Lublin	WolicaPierwsza1	<LOD	0.026	<LOD	0.018	<LOD	<LOD	0.047	medium
53	Lublin	WolicaPierwsza2	<LOD	0.072	0.009	0.050	<LOD	<LOD	0.140	high
54	Lublin	WolicaPierwsza3	<LOD	0.014	0.007	0.076	<LOD	<LOD	0.099	medium
55	Lublin	WolicaPierwsza4	<LOD	0.021	<LOD	0.011	<LOD	<LOD	0.034	medium
56	Lublin	WolicaPierwsza5	<LOD	0.005	<LOD	<LOD	<LOD	<LOD	0.006	low
57	Lublin	Zamość	<LOD	0.203	<LOD	0.0193	<LOD	<LOD	0.246	high
58	Lubusz	Dąbrowiec	<LOD	<LOD	<LOD	<LOD	<LOD	<LOD	<LOD	no
59	Lubusz	Słubice1	<LOD	0.010	<LOD	0.013	<LOD	<LOD	0.024	low

Sample number	Voivodeship	Localization	o,p'-DDE	p,p'-DDE	p,p'-DDD	p,p'-DDT	o,p'-DDT	o,p'-DDD	∑DDT concentration	Contamination category
			detection limit 0.005 mg/kg							
60	Lubusz	Słubice2	<LOD	0.017	0.012	0.031	<LOD	<LOD	0.063	medium
61	Lubusz	Słubice3	<LOD	0.021	0.007	0.012	<LOD	<LOD	0.043	medium
62	Lubusz	Wichów	<LOD	0.007	<LOD	0.006	<LOD	<LOD	0.014	low
63	Łódź	Brójce	<LOD	0.070	<LOD	0.016	<LOD	<LOD	0.093	medium
64	Łódź	DębowaGóra1	<LOD	<LOD	<LOD	0.008	<LOD	<LOD	0.008	low
65	Łódź	DębowaGóra2	<LOD	0.037	0.025	0.126	<LOD	<LOD	0.194	high
66	Łódź	DębowaGóra3	<LOD	0.030	0.017	0.025	<LOD	<LOD	0.077	medium
67	Łódź	Godzianów1	<LOD	<LOD	<LOD	<LOD	<LOD	<LOD	<LOD	no
68	Łódź	Godzianów2	<LOD	<LOD	<LOD	<LOD	<LOD	<LOD	<LOD	no
69	Łódź	Godzianów3	<LOD	<LOD	<LOD	<LOD	<LOD	<LOD	<LOD	no
70	Łódź	Godzianów4	<LOD	<LOD	<LOD	<LOD	<LOD	<LOD	<LOD	no
71	Łódź	Ignacew	<LOD	0.022	0.012	0.026	<LOD	<LOD	0.064	medium
72	Łódź	Niemirowice	<LOD	<LOD	<LOD	<LOD	<LOD	<LOD	<LOD	no
73	Łódź	NoweRowiska	<LOD	0.040	0.012	0.051	<LOD	<LOD	0.109	medium
74	Łódź	NoweSzwejki	<LOD	0.018	0.008	0.010	<LOD	<LOD	0.039	medium
75	Łódź	NowyDwór-Parcela	<LOD	<LOD	<LOD	<LOD	<LOD	<LOD	<LOD	no
76	Łódź	Rossocha	<LOD	0.016	0.0182	0.0609	<LOD	<LOD	0.099	medium
77	Łódź	RudaBugaj	<LOD	<LOD	<LOD	<LOD	<LOD	<LOD	<LOD	no
78	Łódź	Skierniewice1	<LOD	0.025	0.0083	0.0203	<LOD	<LOD	0.057	medium
79	Łódź	Skierniewice2	<LOD	0.005	<LOD	0.007	<LOD	<LOD	0.013	low
80	Łódź	Skierniewice3	<LOD	0.028	0.014	0.011	<LOD	<LOD	0.058	medium
81	Łódź	Skierniewice4	<LOD	0.023	<LOD	<LOD	<LOD	<LOD	0.026	medium
82	Łódź	Suliszew	<LOD	<LOD	<LOD	<LOD	<LOD	<LOD	<LOD	no
83	Łódź	Trzcianna	<LOD	<LOD	<LOD	<LOD	<LOD	<LOD	<LOD	no
84	Łódź	Wysokienice	<LOD	<LOD	<LOD	<LOD	<LOD	<LOD	<LOD	no
85	Łódź	Zamoście1	<LOD	0.009	<LOD	<LOD	<LOD	<LOD	0.010	low
86	Łódź	Zamoście2	<LOD	0.011	0.026	0.057	<LOD	<LOD	0.098	medium
87	Łódź	Żelazna	<LOD	<LOD	<LOD	0.0088	<LOD	<LOD	0.009	low
88	Masovian	Czarna	<LOD	0.009	<LOD	0.015	<LOD	<LOD	0.024	low
89	Masovian	Hołowienki1	<LOD	<LOD	<LOD	<LOD	<LOD	<LOD	<LOD	no

Sample number	Voivodeship	Localization	o,p'-DDE	p,p'-DDE	p,p'-DDD	p,p'-DDT	o,p'-DDT	o,p'-DDD	∑DDT concentration	Contamination category
			detection limit 0.005 mg/kg							
90	Masovian	Hołowienki2	<LOD	<LOD	<LOD	<LOD	<LOD	<LOD	<LOD	no
91	Masovian	Kamion1	<LOD	0.017	<LOD	<LOD	<LOD	<LOD	0.019	low
92	Masovian	Kamion2	<LOD	0.028	0.008	0.025	<LOD	<LOD	0.065	medium
93	Masovian	Kańkowo	<LOD	0.260	0.081	<LOD	<LOD	<LOD	0.380	high
94	Masovian	KoloniaHołowienki	<LOD	0.007	<LOD	0.005	<LOD	<LOD	0.013	low
95	Masovian	Kozłówk.Radomia	<LOD	<LOD	<LOD	<LOD	<LOD	<LOD	<LOD	no
96	Masovian	Krukowo	<LOD	0.300	0.031	<LOD	<LOD	0.013	0.383	high
97	Masovian	Michałowice	<LOD	0.005	<LOD	<LOD	<LOD	<LOD	0.006	low
98	Masovian	Modrzewina	<LOD	0.025	0.015	0.053	<LOD	<LOD	0.097	medium
99	Masovian	Mogielnica	<LOD	0.048	0.028	0.120	<LOD	<LOD	0.205	high
100	Masovian	NowyOryszew	<LOD	<LOD	<LOD	<LOD	<LOD	<LOD	<LOD	no
101	Masovian	Radzanów	<LOD	0.013	<LOD	0.007	<LOD	<LOD	0.022	low
102	Masovian	Rębisze-Parcele	<LOD	0.009	<LOD	0.007	<LOD	<LOD	0.017	low
103	Masovian	StaryBoguszyn	<LOD	0.007	<LOD	0.012	<LOD	<LOD	0.020	low
104	Masovian	Śniadowo1	<LOD	0.010	<LOD	0.024	<LOD	<LOD	0.034	medium
105	Masovian	Śniadowo2	<LOD	0.017	0.020	0.258	<LOD	<LOD	0.299	high
106	Masovian	Śniadowo3	<LOD	0.023	0.008	0.043	<LOD	<LOD	0.078	medium
107	Masovian	Tchórzenica	<LOD	0.015	0.010	0.022	<LOD	<LOD	0.049	medium
108	Opole	Biadacz-Kamienisko	<LOD	0.018	<LOD	0.008	<LOD	<LOD	0.028	medium
109	Opole	Szczedrzyk	<LOD	0.037	<LOD	0.039	<LOD	<LOD	0.080	medium
110	Podlaskie	Białousy1	<LOD	0.006	<LOD	0.02	<LOD	<LOD	0.026	medium
111	Podlaskie	Białousy2	<LOD	0.007	<LOD	<LOD	<LOD	<LOD	0.008	low
112	Podlaskie	Jastrzębna1	<LOD	0.006	<LOD	<LOD	<LOD	<LOD	0.007	low
113	Podlaskie	Jastrzębna2	<LOD	<LOD	<LOD	<LOD	<LOD	<LOD	<LOD	no
114	Pomeranian	Cetyń	<LOD	0.017	<LOD	0.021	<LOD	<LOD	0.04	medium
115	Pomeranian	Nakla	<LOD	<LOD	<LOD	<LOD	<LOD	<LOD	<LOD	no
116	Pomeranian	Przezmark	<LOD	0.033	<LOD	0.010	<LOD	<LOD	0.047	medium
117	Pomeranian	Sycewice	<LOD	0.100	0.044	<LOD	<LOD	<LOD	0.160	high
118	Subcarpathian	WolaOtałęska	<LOD	0.009	<LOD	0.012	<LOD	<LOD	0.022	low
119	Subcarpathian	Zalesie	<LOD	0.008	<LOD	0.01	<LOD	<LOD	0.019	low

Sample number	Voivodeship	Localization	o,p'-DDE	p,p'-DDE	p,p'-DDD	p,p'-DDT	o,p'-DDT	o,p'-DDD	∑DDT concentration	Contamination category
			detection limit 0.005 mg/kg							
120	Świętokrzyskie	Boria	<LOD	0.017	<LOD	0.008	<LOD	<LOD	0.027	medium
121	Warmian-Masurian	Spytajny	<LOD	0.015	0.007	0.033	<LOD	<LOD	0.057	medium
122	West Pomeranian	Czaplinek	<LOD	0.177	0.035	0.093	0.031	0.009	0.370	high
123	West Pomeranian	Dolice1	<LOD	0.093	0.041	0.099	0.021	0.006	0.275	high
124	West Pomeranian	Dolice2	<LOD	0.015	<LOD	0.026	<LOD	<LOD	0.043	medium
125	West Pomeranian	Łąka1	<LOD	0.005	<LOD	<LOD	<LOD	<LOD	0.006	low
126	West Pomeranian	Łąka2	<LOD	0.026	0.005	0.020	<LOD	<LOD	0.055	medium
127	West Pomeranian	Łąka3	<LOD	0.055	0.007	0.027	<LOD	<LOD	0.095	medium
128	West Pomeranian	Modrzewo	<LOD	0.007	<LOD	<LOD	<LOD	<LOD	0.008	low
129	West Pomeranian	Radomyśl	<LOD	0.008	<LOD	<LOD	<LOD	<LOD	0.009	low
130	West Pomeranian	Stepniczka	<LOD	0.072	0.139	<LOD	<LOD	0.0306	0.268	high
131	West Pomeranian	Tarnowo1	<LOD	0.007	<LOD	0.006	<LOD	<LOD	0.014	low
132	West Pomeranian	Tarnowo2	<LOD	0.008	<LOD	0.005	<LOD	<LOD	0.014	low
133	West Pomeranian	Tarnowo3	<LOD	0.047	0.013	0.040	<LOD	<LOD	0.106	medium
134	West Pomeranian	Tarnowo4	<LOD	0.063	0.010	0.030	<LOD	<LOD	0.111	medium
135	West Pomeranian	Tarnowo5	<LOD	0.053	0.009	0.045	<LOD	<LOD	0.114	medium
136	West Pomeranian	Tarnowo6	<LOD	0.020	<LOD	0.008	<LOD	<LOD	0.030	medium
137	West Pomeranian	Tarnowo7	<LOD	0.110	0.019	0.069	<LOD	<LOD	0.212	high
138	West Pomeranian	Tarnowo8	<LOD	0.029	0.005	0.012	<LOD	<LOD	0.051	medium
139	West Pomeranian	Tarnowo9	<LOD	0.026	0.007	0.017	<LOD	<LOD	0.054	medium
140	West Pomeranian	Wołczkowo1	<LOD	0.037	0.011	0.033	<LOD	<LOD	0.087	medium
141	West Pomeranian	Wołczkowo2	<LOD	0.020	0.006	0.011	<LOD	<LOD	0.039	medium
142	West Pomeranian	Żabówko	<LOD	0.029	0.023	<LOD	<LOD	0.0061	0.064	medium
