# Supplementary material for: Monitoring of DDT in Agricultural Soils under Organic Farming in Poland and the Risk of Crop Contamination
Source: Environ Manage. 2020 Aug 19;66(5):916–29. doi: 10.1007/s00267-020-01347-9 (PMC7591450; doi:10.1007/s00267-020-01347-9)
Supplement: Supplementary file 2 — Supplementary Table S2 [file 267_2020_1347_MOESM2_ESM.pdf]

Table EMS2. Total DDT concentration, history of field use and pH of soil samples among those monitored. Samples number corresponds to those in Table EMS1.

Sample number	Voivodeship	Localization	GPS coordinates	Soil type*	pH	∑DDT concentration	Prior use of the field
2	Greater Poland	Lubień1	52.398974, 15.993624	Soil in early development, loamy, sandy-loamy	5.14	0.012	Managed according to organic farming since 2010; previously conventional annual crops and a period of set aside
3	Greater Poland	Lubień2			4.34	0.022	
8	Greater Poland	Wiry	52.322670, 16.855194	Brown, leached and precipitation-gley soils, loamy or loamy sands	6.87	0.042	Managed according to organic farming since 2005; previously long-term fruit crops
15	Lesser Poland	Goszyce	50.175416, 20.127173	Proper and leached brown soils developed from loess and loess-like formations	6.14	0.089	Managed according to organic farming since 2009; previously long-term fruit and vegetable crops
16	Lesser Poland	Maszkowice1	49.549808, 20.466128	Acid and leached brown soils produced from weathered rocks	5.39	0.009	Managed according to organic farming since 2005; previously long-term fruit crops
17	Lesser Poland	Maszkowice2	49.552178, 20.468113		6.16	0.015	
18	Lower Silesian	Biestrzyków	51.027213, 17.039131	Leached acidic brown soils, sandy clay loam	6.09	0.106	Managed according to organic farming since 2005; previously long-term agricultural crops
19	Lower Silesian	Chocieszów1	50.462815, 16.462585	Soil early in development, rocky or acid and leached brown soils produced from weathered rocks	5.13	0.058	Managed according to organic farming since 2000; previously long-term fruit crops
20	Lower Silesian	Chocieszów2			4.95	0.034	
21	Lower Silesian	Lipiany1	51.343640, 15.633149	Brown, leached and precipitation-gley soils, loamy or loamy sands	5.63	0.030	Managed according to organic farming since 2002; previously long-term vegetable crops grown using a pro-organic system
22	Lower Silesian	Lipiany2			5.50	0.042	
62	Lubusz	Brzeźnica	51.752069, 15.467233	Brown, leached and precipitation-gley soils, loamy or loamy sands	4.16	0.014	Managed according to organic farming since 2008; previously land owned by other owners, probably intensive cereal and agricultural crops

Sample number	Voivodeship	Localization	GPS coordinates	Soil type*	pH	∑DDT concentration	Prior use of the field
23	Lublin	Andrzejów	50.758398, 22.437402	Brown, leached and precipitation-gley soils, loamy or loamy sands from loess and loess-like formations	4.33	0.089	Managed according to organic farming since 2000; previously used for intensive crops
31	Lublin	GrabówRycki1	51.685552, 22.090279	Soils produced from loamy or loamy sandy	6.62	0.025	Managed according to organic farming since 2006; previously long-term cereal crops
32	Lublin	GrabówRycki2			6.73	0.026	
33	Lublin	GrabówRycki3			4.90	0.055	
37	Lublin	KarczmiskaDrugie1	51.242740, 22.040703	Brown, leached and precipitation-gley soils, produced from loess and loess-like formations	5.08	0.035	Managed according to organic farming since 2000; previously used intensively for annual and fruit crops
38	Lublin	KarczmiskaDrugie2			5.77	0.010	
39	Lublin	Hałasy1	52.035600, 22.742688	Brown, leached and precipitation-gley soils, loamy or loamy sands	7.33	0.011	Under conversion to organic farming since 2016; previously long-term different annual, vegetable and fruit crops
40	Lublin	Hałasy2			7.26	0.014	
46	Lublin	Przestrzeń1	51.624980, 22.148413	Brown, leached and precipitation-gley soils, loamy or loamy sands	4.92	0.039	Managed according to organic farming since 2009; previously intensive annual crops
47	Lublin	Przestrzeń2			4.93	0.022	
53	Lublin	WolicaPierwsza2	50.762008, 22.400730	Ferric soils produced from loam, loamy and loose sands	5.08	0.140	Managed according to organic farming since 2000; previously long-term annual and vegetable crops
54	Lublin	WolicaPierwsza3			4.45	0.099	
55	Lublin	WolicaPierwsza4			5.03	0.034	
56	Lublin	WolicaPierwsza5			5.87	0.006	
73	Łódź	NoweRowiska	51.895815, 20.118019	Brown, leached and precipitation-gley soils, loamy or loamy sands	6.06	0.109	Managed according to organic farming since 2007; previously long-term fruit crops (since around 1950)
85	Łódź	Zamoście1	51.131304, 19.169251	Gley and mineral soils	5.25	0.010	Managed according to organic farming since 2004; previously long-term cereal crops and potatoes
86	Łódź	Zamoście2			5.29	0.098	

Sample number	Voivodeship	Localization	GPS coordinates	Soil type*	pH	∑DDT concentration	Prior use of the field
88	Masovian	Czarna	52.521736, 20.615578	Brown, leached and precipitation-gley soils, loamy or loamy sands	5.75	0.024	Managed according to organic farming since 2012; previously intensive long-term cereal crops
97	Masovian	Michałowice	51.632225, 20.724792	Brown, leached and precipitation-gley soils, loamy or loamy sands	5.47	0.006	Managed according to organic farming since 2005; previously long-term fruit crops
98	Masovian	Modrzewina	51.735668, 20.802997	Brown, leached and precipitation-gley soils, loamy or loamy sands	4.89	0.097	Managed according to organic farming since 2012; previously long-term fruit crops
99	Masovian	Mogielnica	51.686649, 20.711668	Ferric soils produced from loam, loamy and loose sands or brown, leached and precipitation-gley soils, loamy or loamy sands	4.17	0.205	Managed according to organic farming since 2007; previously long-term fruit crops
102	Masovian	Rębisze-Parcele	52.928280, 21.558095	Brown, leached and precipitation-gley soils, loamy or loamy sands	7.36	0.017	Managed according to organic farming since 2014; previously long-term fruit and vegetable crops
104	Masovian	Śniadowo1	52.515688, 20.625926	Brown, leached and precipitation-gley soils, loamy or loamy sands	5.11	0.034	Managed according to organic farming since 2007; previously long-term cereal crops
105	Masovian	Śniadowo2			5.44	0.299	
106	Masovian	Śniadowo3			6.06	0.078	
108	Opole	Biadacz-Kamienisko	51.005631, 18.352010	Ferric soils produced from loam, loamy and loose sands	4.16	0.028	Managed according to organic farming since 1996; previously vegetable crops and long-term annual crops

Sample number	Voivodeship	Localization	GPS coordinates	Soil type*	pH	∑DDT concentration	Prior use of the field
109	Opole	Szczedrzyk	50.694342, 18.178799	River silty soils made of sand, clay, or silt and clay	5.02	0.080	Managed according to organic farming since 2004; previously fruit crops (since around 1966) and annual crops
118	Subcarpathian	WolaOtałęska	50.350094, 21.260944	Ferric soils produced from loam, loamy and loose sands or river silts made of sand, clay, silt and clay	6.90	0.022	Managed according to organic farming since 2012; previously long-term cereal crops
119	Subcarpathian	Zalesie	50.141078, 23.000224	Gley and mineral soils	5.21	0.019	Managed according to organic farming since 2005; previously cereal crops
116	Pomeranian	Przezmark	53.856098, 19.512968	Leached acidic brown soils, sandy, clay and loamy	7.38	0.047	Managed according to organic farming since 2001; previously probably long-term cereal crops
120	Świętokrzyskie	Boria	50.977972, 21.537766	Brown, leached and precipitation-gley soils, loamy or loamy sands	6.90	0.027	Managed according to organic farming since 2012; previously intensive annual and vegetable crops
121	Warmian-Masurian	Spytajny	54.251410, 20.766852	Proper and leached brown soils, clay sandy, clay, and aquatic deposits and clays	4.95	0.057	Managed according to organic farming since 2005; previously long-term cereal crops
124	West Pomeranian	Dolice	53.204704, 15.206664	Proper and leached brown soils, clay sandy, clay, and aquatic deposits and clays	5.22	0.043	Managed according to organic farming since 2006; previously long-term annual crops
125	West Pomeranian	Łąka1	53.541053, 16.200944	Brown, leached and precipitation-gley soils, loamy or loamy sands	5.21	0.006	Managed according to organic farming since 2005; previously long-term cereal crops
126	West Pomeranian	Łąka2	53.540530, 16.207650		4.03	0.055	
127	West Pomeranian	Łąka3			4.39	0.095	
128	West Pomeranian	Modrzewo	53.308028, 15.388458	Brown, leached and precipitation-gley soils, loamy or loamy sands	4.20	0.008	Managed according to organic farming since 2014; previously long-term cereal and vegetable crops

Sample number	Voivodeship	Localization	GPS coordinates	Soil type*	pH	∑DDT concentration	Prior use of the field
129	West Pomeranian	Radomyśl	53.758099, 16.534005	Ferric soils produced from loam, loamy and loose sands	5.30	0.009	Managed according to organic farming since 2014; previously long-term fruit crops
131	West Pomeranian	Tarnowo1	53.311207, 15.357271	Brown, leached and precipitation-gley soils, loamy or loamy sands	4.66	0.014	Managed according to organic farming since 2013; previously long-term cereal and vegetable crops
132	West Pomeranian	Tarnowo2			4.52	0.014	
133	West Pomeranian	Tarnowo3			3.91	0.106	
134	West Pomeranian	Tarnowo4			5.00	0.111	
135	West Pomeranian	Tarnowo5			4.39	0.114	
136	West Pomeranian	Tarnowo6			4.71	0.030	
137	West Pomeranian	Tarnowo7			4.12	0.212	
138	West Pomeranian	Tarnowo8			4.25	0.051	
139	West Pomeranian	Tarnowo9			4.68	0.054	
140	West Pomeranian	Wołczkowo1	53.478016, 14.445167	River silts made of sand, clay, silt and clay	4.96	0.087	Managed according to organic farming since 2005; previously pastures and fallow before growing vegetable crops and herbs
141	West Pomeranian	Wołczkowo2			6.18	0.039	

* Based on: Uggla H., Uggla Z. 1976. Gleboznawstwo leśne. Mapa gleb Polski. PWRiL. Warsaw, Poland
